# Computer vision applied to dual-energy computed tomography images for precise calcinosis cutis quantification in patients with systemic sclerosis

**DOI:** 10.1186/s13075-020-02392-9

**Published:** 2021-01-06

**Authors:** Anita C. Chandrasekaran, Zhicheng Fu, Reid Kraniski, F. Perry Wilson, Shannon Teaw, Michelle Cheng, Annie Wang, Shangping Ren, Imran M. Omar, Monique E. Hinchcliff

**Affiliations:** 1 Yale School of Medicine, Section of Rheumatology, Allergy & Immunology, The Anlyan Center, 300 Cedar Street, PO BOX 208031, New Haven, CT 06520 USA; 2 Department of Computer Science, Illinois Institute of Technology, 10 W 31st St, Chicago, IL 60616 USA; 3 Motorola Mobility LLC, 222 W Merchandise Mart Plaza #1800, Chicago, IL 60654 USA; 4 Department of Radiology, Yale School of Medicine, 330 Cedar St, New Haven, CT 06520 USA; 5 Clinical and Translational Research Accelerator, Department of Medicine, Yale School of Medicine, Temple Medical Center, 60 Temple Street Suite 6C, New Haven, CT 06510 USA; 6 Department of Computer Science, San Diego State University, 5500 Campanile Drive, San Diego, CA 92182 USA; 7 Department of Radiology, Northwestern University Feinberg School of Medicine, 676 N St Clair St, Chicago, IL 60611 USA; 8 Department of Medicine, Division of Rheumatology, Northwestern University Feinberg School of Medicine, 240 E. Huron Street, Suite M-300, Chicago, IL 60611 USA

**Keywords:** Systemic sclerosis, Scleroderma, Calcinosis cutis, Computer vision, Convolutional neural networks (CNN), Dystrophic calcifications, U-Net, Artificial intelligence, Medical image analysis

## Abstract

**Background:**

Although treatments have been proposed for calcinosis cutis (CC) in patients with systemic sclerosis (SSc), a standardized and validated method for CC burden quantification is necessary to enable valid clinical trials. We tested the hypothesis that computer vision applied to dual-energy computed tomography (DECT) finger images is a useful approach for precise and accurate CC quantification in SSc patients.

**Methods:**

De-identified 2-dimensional (2D) DECT images from SSc patients with clinically evident lesser finger CC lesions were obtained. An expert musculoskeletal radiologist confirmed accurate manual segmentation (subtraction) of the phalanges for each image as a gold standard, and a U-Net Convolutional Neural Network (CNN) computer vision model for segmentation of healthy phalanges was developed and tested. A validation study was performed in an independent dataset whereby two independent radiologists manually measured the longest length and perpendicular short axis of each lesion and then calculated an estimated area by assuming the lesion was elliptical using the formula long axis/2 × short axis/2 × *π*, and a computer scientist used a region growing technique to calculate the area of CC lesions. Spearman’s correlation coefficient, Lin’s concordance correlation coefficient with 95% confidence intervals (CI), and a Bland-Altman plot (Stata V 15.1, College Station, TX) were used to test for equivalence between the radiologists’ and the CNN algorithm-generated area estimates.

**Results:**

Forty de-identified 2D DECT images from SSc patients with clinically evident finger CC lesions were obtained and divided into training (*N* = 30 with image rotation × 3 to expand the set to *N* = 120) and test sets (*N* = 10). In the training set, five hundred epochs (iterations) were required to train the CNN algorithm to segment phalanges from adjacent CC, and accurate segmentation was evaluated using the ten held-out images. To test model performance, CC lesional area estimates calculated by two independent radiologists and a computer scientist were compared (radiologist 1 vs. radiologist 2 and radiologist 1 vs. computer vision approach) using an independent test dataset comprised of 31 images (8 index finger and 23 other fingers). For the two radiologists’, and the radiologist vs. computer vision measurements, Spearman’s rho was 0.91 and 0.94, respectively, both *p* < 0.0001; Lin’s concordance correlation coefficient was 0.91 (95% CI 0.85–0.98, *p* < 0.001) and 0.95 (95% CI 0.91–0.99, *p* < 0.001); and Bland-Altman plots demonstrated a mean difference between radiologist vs. radiologist, and radiologist vs. computer vision area estimates of − 0.5 mm^2^ (95% limits of agreement − 10.0–9.0 mm^2^) and 1.7 mm^2^ (95% limits of agreement − 6.0–9.5 mm^2^, respectively.

**Conclusions:**

We demonstrate that CNN quantification has a high degree of correlation with expert radiologist measurement of finger CC area measurements. Future work will include segmentation of 3-dimensional (3D) images for volumetric and density quantification, as well as validation in larger, independent cohorts.

## Background

Calcinosis cutis (CC) is defined as the deposition of insoluble calcium salts within the skin and subcutaneous tissues [1]. There are five subtypes of calcinosis cutis: dystrophic, metastatic, iatrogenic, calciphylaxis, and idiopathic [1]. Dystrophic calcification from local tissue injury is the most common subtype and is associated with autoimmune disorders including systemic sclerosis (SSc) and juvenile dermatomyositis (JDM). The clinical presentation is highly variable including an incidental finding on imaging, lesions in the hands or pressure point areas resulting in soft tissue swelling and ulceration, to disabling and life-threatening lesions [1]. Upwards of 18–49% of SSc patients [1], 40% of JDM patients [2], and 1% of chronic renal dialysis patients [3] experience CC.

Calcinosis cutis remains a therapeutic challenge. Medications for CC include calcium channel blockers (e.g., diltiazem), warfarin, topical and intravenous sodium thiosulfate and aluminum hydroxide (chelation therapy), ceftriaxone, minocycline, colchicine, intravenous immunoglobulin (IVIg), and probenecid among others [1]. The data supporting the efficacy of these treatments are primarily from case reports, case series, and retrospective studies rather than randomized clinical trials [1]. Valid clinical trial design to test the safety and efficacy of proposed systemic treatments requires a feasible, precise, and reproducible method for CC burden quantification.

Dual-energy computed tomography (DECT), also known as “spectral imaging” [4], is an imaging modality established for the detection of coronary calcifications [5] and uric acid nephrolithiasis [6], and has more recently been studied for the identification and quantification of soft tissue monosodium urate deposits in patients with gout [7]. Single-energy computed tomography (CT) uses a single polychromatic X-ray beam, which is emitted and received from a single source and detector, respectively [8]. This limits its use in the differentiation of materials (e.g., fat, soft tissues) that have similar linear attenuation coefficients [8]. In contrast, DECT utilizes two energy levels [(usually 80 and 140 kilovoltage peaks (kVp)] [4], that results in greater differentiation between attenuation coefficients [9] and is particularly efficient for the evaluation of small body parts including the extremities [8]. Dual-energy computed tomography permits differentiation of CC lesions from adjacent bone, but radiologists must manually quantify lesional CC areas, which is time-consuming and therefore costly, and prone to inter-rater variability. Moreover, CC lesions are often irregularly shaped, which can lead to imprecise and inaccurate measurements. By convention, CC lesions are measured in three planes, using the maximal dimension as the reference axis, with two additional orthogonal axes. Using the ellipsoid formula, utilized in other clinical applications such as prostate measurement, a multiplication factor of 0.52 can then be incorporated for volume estimation [10, 11]. On subsequent exams, lesions need to be measured using the same axes, to maintain consistency and accuracy.

**Fig. 1:**
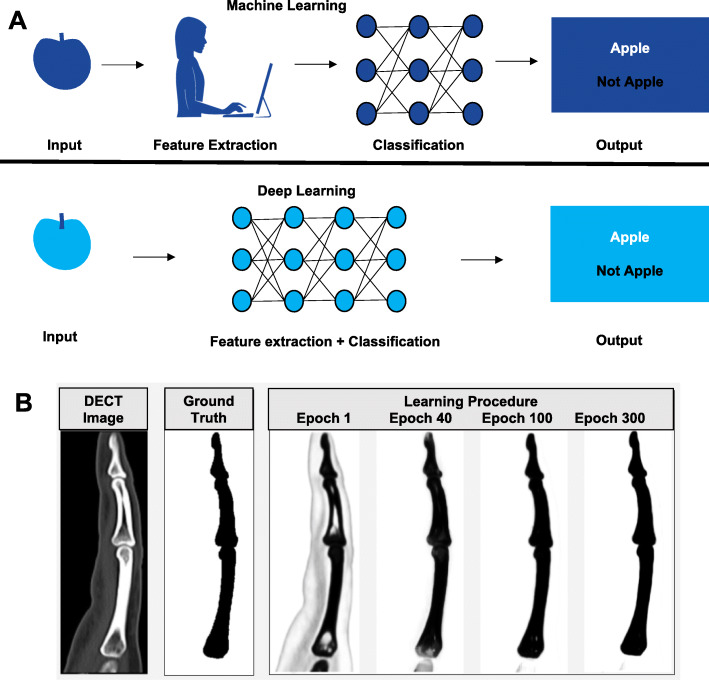
**a** Distinction between machine learning and deep learning. **b** Optimization of the CNN algorithm for forefinger bone segmentation

Computer vision, a field in which computers are trained to independently identify and process images [12], has been increasingly successfully applied to solve problems in clinical medicine. Since the late 1990s, computer scientists have been using large quantities of curated images to develop software systems that enable computers to perform tasks that previously required an expert such as skin disease diagnoses [13–15]. Deep learning methods (e.g., convolutional neural networks [CNN], deep neural networks [DNN], and support vector machines [SVM]) are a more complex form of machine learning and have been developed to enable computers to filter inputs, such as images, sequentially through layers in order to learn how to predict and classify information without manual feature extraction (Fig. 1a) [12]. The process of filtering images using layers whereby the output of one layer becomes the input of a subsequent layer is the same process that neurons in the human brain use for image classification (e.g., car or clock), hence deep learning models are called neural networks [16].

Because large curated training data sets are not available for most diseases, a process called transfer learning was developed to enable algorithms that were developed for a disease with high prevalence to be applied to more rare diseases like SSc-CC [17]. Herein, we optimized a deep learning model using a CNN approach and applied this technology to DECT images of fingers from patients with SSc and clinically evident CC. The goal was to train a computer to quantify CC area lesions that currently requires a highly trained musculoskeletal radiologist. The use of artificial intelligence for accurate, precise, and rapid quantification of CC burden in patients with SSc will facilitate musculoskeletal radiologists’ workflow and improve clinical trial design.

## Methods

### Identification of DECT images containing calcinosis cutis in patients with systemic sclerosis

We conducted a retrospective study in patients with SSc and clinically evident finger CC lesions who were referred for 2-dimensional (2D) DECT imaging. Because no personal health information was collected and all images were de-identified, the study was not considered human subjects research and was exempt from institutional board approval. The Northwestern Memorial Hospital electronic health record was queried for patients with a diagnosis of SSc (ICD9 = 710.1 or ICD10 = M34.9) and DECT hand images. De-identified sagittal reformatted images from patients were downloaded from the Picture Archiving and Communication System (PACS) in *jpeg* form to a secure server maintained by the Northwestern University Information Technology, then transferred to Visage Imaging 7 (San Diego, CA) for examination purposes. All images were optimized to assess bone (window of 1500 and level of 450). Because bone and CC lesions both consist of basic calcium hydroxyapatite, a computer scientist manually segmented (identified) finger phalanges on training set images and an expert musculoskeletal radiologist (IMO) confirmed measurements that were then used as a gold standard reference (ground truth) for the deep learning models (Fig. 1).

### CNN software training

Various segmentation algorithms (e.g., expectation maximization [18], Fuzzy c-means [19], K-means [20], mean-shift segmentation [21], V-Net [22], P-Net [23], and U-Net [24]) were tested in order to identify the optimal approach for finger bone segmentation. U-Net CNN performed best and was utilized for the experiments herein.

### Training set and test set one

Forty sagittal reformatted DECT finger images from three unique patients were divided into a training set (*N* = 30) and a test set (*N* = 10). To enlarge the training dataset to permit development of a U-Net CNN model, 30 images were spatially rotated three times for a total of 90 additional images (total 120 images). A NVIDIA Tesla k40c GPU computing processor with varying numbers of epochs was used (Fig. 1). Visual inspection was conducted between runs to determine the concordance between manual and computer bone segmentation in order to identify the optimal number of epochs. The segmentation approach was tested in ten independent images and manual inspection confirmed segmentation accuracy.

### Calcinosis cutis area estimates

Thirty-one sagittal reformatted DECT finger images containing CC from 13 unique patients were identified as an independent test. The CNN finger bone segmentation algorithm was applied, arithmetic operations were applied to segmented images to subtract out the normal phalanges, and region growing, a pixel-based image segmentation method, was deployed to quantify the area of residual CC lesions [25]. Concurrently, a musculoskeletal radiologist (IMO) measured each lesion’s maximal long axis and maximal perpendicular short axis (mm) and then calculated an estimated area of each lesion by assuming the lesion was elliptical and using the formula long axis/2 × short axis/2 × *π*. A second expert musculoskeletal radiologist (AW) independently measured the area of each CC lesion, and the concordance between radiologists’ measurements was assessed.

### Statistics

First, CC lesional area estimates were compared between radiologists (IMO and AW) to test the precision of area estimates. Next, the radiologist (IMO)- and CNN-generated CC area estimates were compared to test the accuracy of the CNN approach. Spearman’s correlation coefficient was used to assess the correlation, and Lin’s concordance correlation coefficient (CCC) was used to test for equivalence, while Bland-Altman plots were used to evaluate agreement. A *p* value≤ 0.05 was considered significant. Stata V 15.1, College Station, TX, was used for analyses.

## Results

### Training the CNN model

**Fig. 2:**
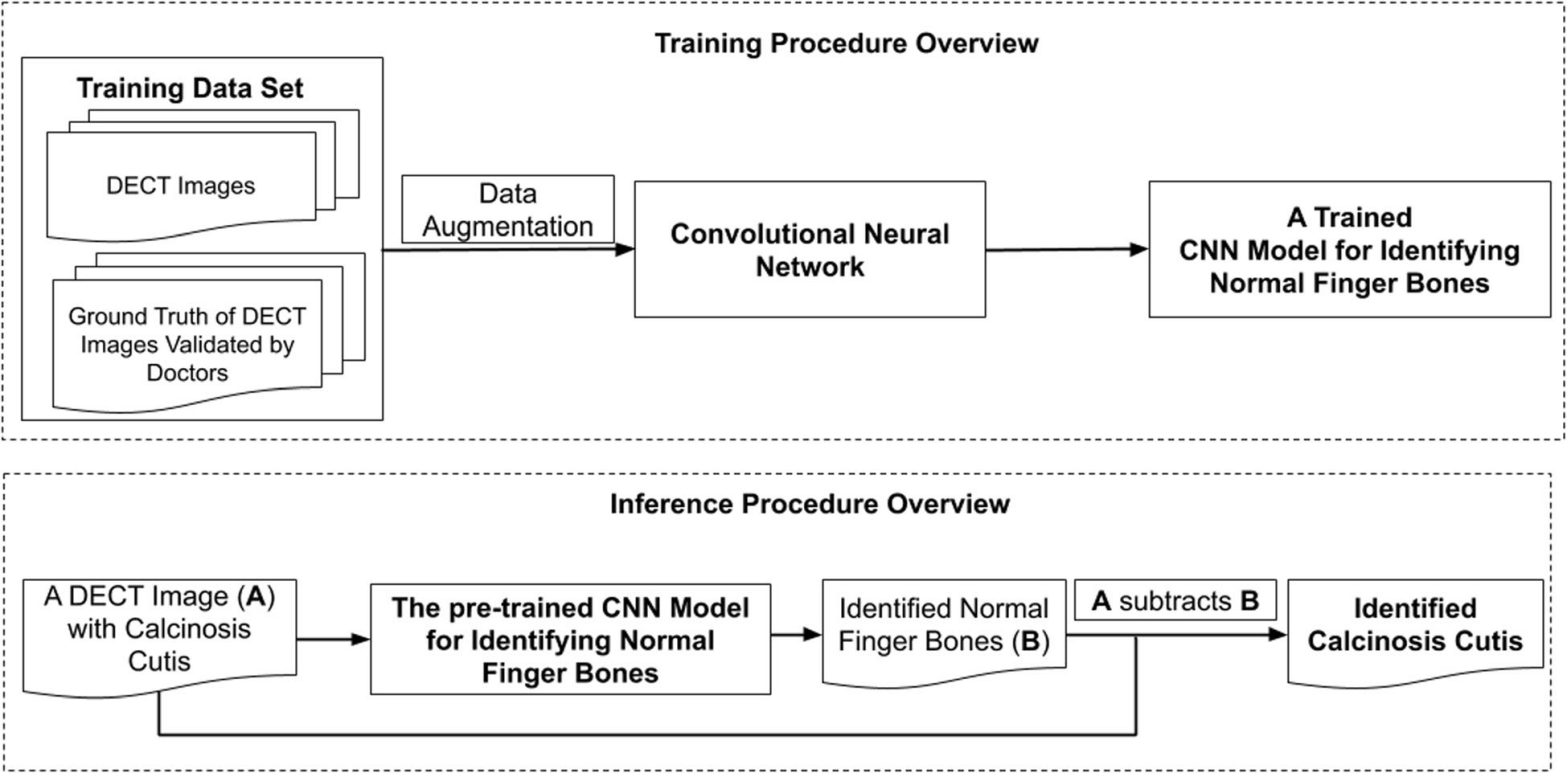
Overview of CNN algorithm training process

**Fig. 3:**
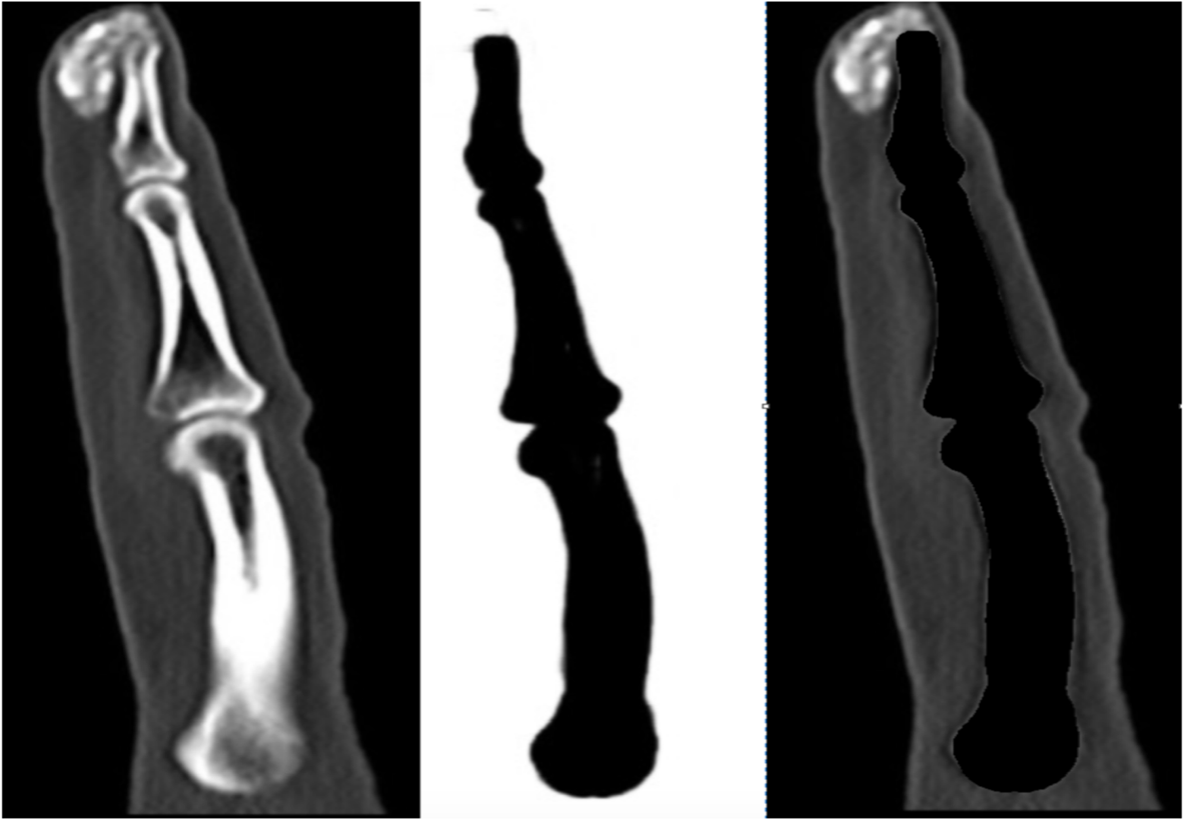
Example of forefinger bone segmentation

The training set (30 images each rotated three times to generate 120 images) was used to train the segmentation algorithm. One hundred, 200, 300, 400, and 500 epochs (iterations) were evaluated and by manual inspection, and 500 epochs was deemed the optimal number (Figs. 1b and 2). A 4-h CNN run time was required for the CNN to learn to discriminate healthy finger bones from adjacent CC lesions (Fig. 3). The algorithm was tested in ten additional DECT finger images, and manual inspection was used to confirm segmentation accuracy.

### Measurement of calcinosis cutis lesions in an independent test set

**Fig. 4:**
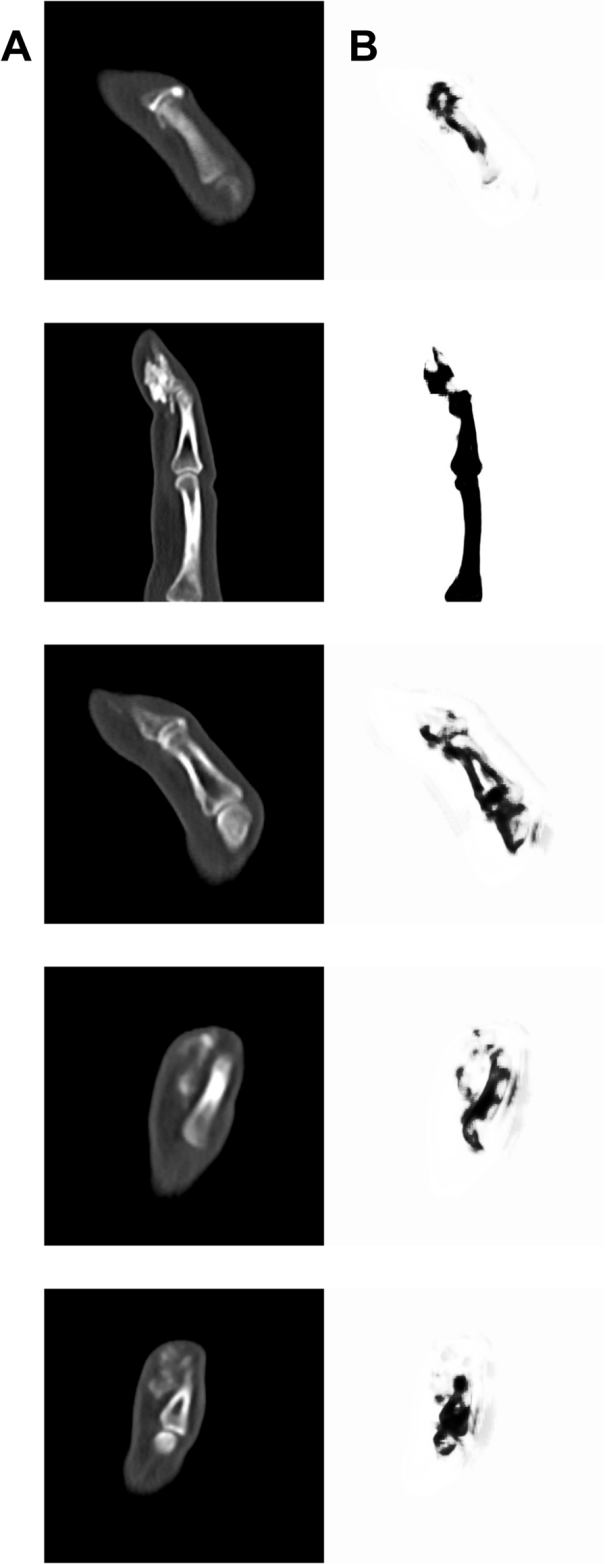
Examples of CC lesions on finger images that were not amenable to U-Net CNN analysis. **a** Dual-energy computed tomography image. **b** U-Net CNN segmentation

Next, the accuracy of the computer vision approach for estimating the CC area was determined in an independent set of 31 images (test set two), by comparing the area estimates generated by computer vision (region growing technique) to those generated by an expert musculoskeletal radiologist. The U-Net CNN algorithm was unable to be applied to seven out of 31 (23%) test set images due to incomplete visualization of the phalanges (Fig. 4). For the 31 images scored by both radiologists and computer vision, the area range for radiologist (IMO) was 0.5–41.5 mm^2^ and for radiologist (AW) was 0.4–40.7 mm^2^. Per the CNN algorithm, 2D area ranged from 0.69–48.18 mm^2^.

**Fig. 5:**
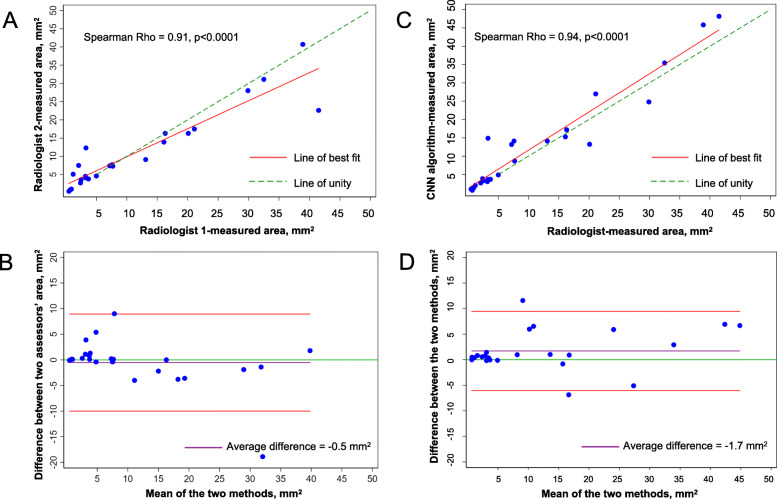
**a** Spearman’s correlation coefficient comparing calcinosis cutis lesional areas measured by **a** two independent radiologists and **c** a radiologist and a computer scientist. Bland-Altman plot, analyzing the agreement between **b** the two independent assessors, and **d** the radiologist’s and computer scientist’s measurement methods. Red lines represent the 95% limits of agreement, purple and green lines represent the mean difference of area measurements between the two methods and line of unity, respectively

The correlation between the two radiologists’ measurements was excellent (Spearman’s rho 0.91, *p* < 0.0001, Lin’s concordance correlation coefficient 0.91 [95% CI 0.85–0.98, *p* < 0.001] (Fig. 5a) and a Bland-Altman plot demonstrating a mean difference − 0.5 mm^2^ (95% limits of agreement − 10.0–9.0 mm^2^) (Fig. 5b). Similarly, the correlation between the radiologist vs. computer vision area estimates was also excellent (Spearman’s rho 0.94, *p* < 0.001), Lin’s concordance correlation coefficient 0.95 [95% CI 0.91–0.99, *p* < 0.001] (Fig. 5c), and a Bland-Altman plot demonstrating a mean difference − 1.7 mm^2^ (95% limits of agreement − 6.0–9.5 mm^2^) (Fig. 5d).

## Discussion

Calcinosis cutis can significantly worsen health-related quality of life, depending on lesion location and size, increasing the risk of infection, and leading to deformities, particularly in patients with SSc [1]. While deep learning technological advancements have improved the performance of image recognition in the field of medical image analysis including the diagnosis of skin diseases [26], computer vision has not been previously applied to the problem of CC quantification. Given a lack of standardized and validated methods to assess CC disease burden, accurate and precise CC quantification represents an unmet medical need. This study evaluated the utility of applying a CNN algorithm to DECT hand images of SSc patients to differentiate CC lesions from adjacent healthy bone and to permit rapid and reproducible quantification of CC lesional area. We found that the computer vision method applied to finger images had a substantial degree of concordance and was highly accurate compared to gold standard radiologist area estimates. These data suggest that computer vision may be a useful approach for CC disease burden quantification.

The difficulty of assessing CC severity in SSc patients has been addressed in prior studies. Utilizing plain X-rays, Chung et al. in 2014 aimed to develop and validate an original scoring system for CC affecting the hands of SSc patients [27]. They developed a feasible scoring system that accounted for lesional area, density, and anatomic location, but significant interrater and intra-rater reliability were noted [27]. In the present study, we used DECT imaging of the hands because this technique has been successfully used to identify and quantify soft tissue monosodium urate deposits in patients with gout [9]. Thus, we hypothesized that DECT would be a useful approach for imaging and quantifying CC in the hands of patients with SSc.

Conventional imaging studies, such as computed tomography (CT) scans and magnetic resonance imaging (MRI), have not been proven to be reliable in the identification of small, irregular calcifications. Newer diagnostic techniques, such as ultrasonography (US), multidetector computed tomography (MDCT), and DECT, have been evaluated in small trials for CC detection [28–31]. A case-control study using US in 44 patients with SSc demonstrated the presence of CC in 17 patients (39%), with a sensitivity of 89% compared to radiography [30]. Multidetector computed tomography has higher tissue resolution and generates 3D images but requires higher radiation exposure and has not been studied in CC [28]. There are minimal current data on the use of DECT in CC. A small study in 2015 of 16 patients with SSc-CC in the hands found that DECT imaging was superior to plain radiographs in localizing soft tissue CC though CC lesions and bone were noted to have the same color and density [7].

We examined the utility of applying CNN, specifically U-Net, to the problem of CC measurement in SSc because U-Net was initially developed for biomedical image segmentation [32]. The two main benefits of U-Net are (1) the ability to develop a useful model in spite of a small training dataset and (2) the U-architecture and skip-connection performance performs better for segmenting different levels of semantic information. The first task to train the computer to “see” the finger bones was readily accomplished in healthy finger bones from SSc patients with normal anatomy. The algorithm failed to recognize the finger bones in cases where the CC lesion was in an area where the entire phalange was not visible (Fig. 4).

Our study has several strengths. Convolutional neural networks have recently been applied for cartilage segmentation on knee MRI scans [33], brain lesion segmentation [34], automatic polyp detection on colonoscopy videos [35], and pulmonary embolism detection on CT [36], but to our knowledge, this is the first study to apply deep learning to analyze CC lesions in SSc patients. Our study involved two expert musculoskeletal radiologists who independently measured CC lesional areas to permit interrater reliability. The computer algorithm was trained to segment the phalanges assessment using real 2D DECT images from SSc patients with clinically evident lesser finger CC lesions and demonstrated a high degree of concordance with a radiologist’s gold standard assessment, despite variable lesional shape and size. This suggests that this deep learning model may be an efficient and reproducible method for CC measurement.

Several limitations are noted as well. This study focused on the hands, specifically lesser finger lesions, because the hands/fingers have been shown to be the area most frequently affected by CC in SSc patients [37]. To be useful in clinical trials, the CNN algorithm will need to be optimized and tested in other anatomic locations. The algorithm can only quantify the area of CC lesions when all three phalanges are visible, limiting its usefulness (Fig. 4). Finally, the principles of 2D segmentation can be transferred to 3-dimensional (3D) segmentation but does require increased computational complexity that was not performed in this study. V-Net is an example of a CNN utilized for 3D segmentation, previously applied to prostate gland segmentation on MRI scans [22], that may be used in future work for CC density quantification, crucial for assessing response to treatment.

## Conclusions

In this study, we demonstrate that computer vision approaches can be applied to the problem of CC quantification in patients with SSc. Expert musculoskeletal radiologist review may not be locally available at many centers, and thus, the ability to add a CNN tool into standard radiology software programs such as the Visage Imaging 7 platform might be advantageous. Convolutional neural network-derived CC quantification had a high degree of correlation with expert radiologist measurement. Moreover, our approach may be generalizable to other diseases that cause dystrophic calcification, such as chronic kidney disease, JDM, and malignancy. To facilitate optimization of computer vision approaches to aid in the quantification of CC, a de-identified dataset of our DECT images has been made publicly available online (https://drive.google.com/drive/folders/1_tXg3dnSQOrZ4_Ro-0JZ3h_hMv0HIe9h?usp=sharing). Future work will involve transferring the principles of 2D segmentation to 3D segmentation for volumetric and density CC quantification as well as additional validation in larger, multi-center SSc cohorts.

## Data Availability

The datasets generated and/or analyzed during the current study will be made publicly available.

## Abbreviations

CC: Calcinosis cutis
SSc: Systemic sclerosis
JDM: Juvenile dermatomyositis
IVIg: Intravenous immunoglobulin
DECT: Dual energy computed tomography
CT: Computed tomography
CNN: Convolutional neural network
SVM: Support vector machine
2D: Two-dimensional
PACS: Picture Archiving and Communication System
CCC: Concordance correlation coefficient
SD: Standard deviation
US: Ultrasound
MDCT: Multidetector-computed tomography
3D: Three-dimensional
